# Pili-like proteins of *Akkermansia muciniphila* modulate host immune responses and gut barrier function

**DOI:** 10.1371/journal.pone.0173004

**Published:** 2017-03-01

**Authors:** Noora Ottman, Justus Reunanen, Marjolein Meijerink, Taija E. Pietilä, Veera Kainulainen, Judith Klievink, Laura Huuskonen, Steven Aalvink, Mikael Skurnik, Sjef Boeren, Reetta Satokari, Annick Mercenier, Airi Palva, Hauke Smidt, Willem M. de Vos, Clara Belzer

**Affiliations:** 1 Laboratory of Microbiology, Wageningen University, Wageningen, The Netherlands; 2 Department of Biosciences, University of Helsinki, Helsinki, Finland; 3 Institute of Environmental Medicine, Karolinska Institutet, Stockholm, Sweden; 4 Cancer and Translational Medicine Research Unit, Biocenter Oulu and Faculty of Medicine, University of Oulu, Oulu, Finland; 5 Host-Microbe Interactomics, Animal Sciences, Wageningen University, Wageningen, The Netherlands; 6 Department Risk Analysis for Products in Development, TNO, Zeist, the Netherlands; 7 Department of Veterinary Biosciences, Faculty of Veterinary Medicine, University of Helsinki, Helsinki, Finland; 8 Pharmacology, Faculty of Medicine, University of Helsinki, Helsinki, Finland; 9 Department of Bacteriology and Immunology, and Research Programs Unit, Immunobiology, University of Helsinki, Helsinki, Finland; 10 Hematology Research Unit Helsinki, University of Helsinki and Department of Hematology, Helsinki University Hospital Comprehensive Cancer Center, Helsinki, Finland; 11 Helsinki University Central Hospital Laboratory Diagnostics, Helsinki, Finland; 12 Laboratory of Biochemistry, Wageningen University, Wageningen, The Netherlands; Instituto de Agroquimica y Tecnologia de Alimentos, SPAIN

## Abstract

Gut barrier function is key in maintaining a balanced response between the host and its microbiome. The microbiota can modulate changes in gut barrier as well as metabolic and inflammatory responses. This highly complex system involves numerous microbiota-derived factors. The gut symbiont *Akkermansia muciniphila* is positively correlated with a lean phenotype, reduced body weight gain, amelioration of metabolic responses and restoration of gut barrier function by modulation of mucus layer thickness. However, the molecular mechanisms behind its metabolic and immunological regulatory properties are unexplored. Herein, we identify a highly abundant outer membrane pili-like protein of *A*. *muciniphila* MucT that is directly involved in immune regulation and enhancement of trans-epithelial resistance. The purified Amuc_1100 protein and enrichments containing all its associated proteins induced production of specific cytokines through activation of Toll-like receptor (TLR) 2 and TLR4. This mainly leads to high levels of IL-10 similar to those induced by the other beneficial immune suppressive microorganisms such as *Faecalibacterium prausnitzii* A2-165 and *Lactobacillus plantarum* WCFS1. Together these results indicate that outer membrane protein composition and particularly the newly identified highly abundant pili-like protein Amuc_1100 of *A*. *muciniphila* are involved in host immunological homeostasis at the gut mucosa, and improvement of gut barrier function.

## Introduction

The human gastrointestinal (GI) tract provides a living environment for the complex and diverse microbiota, which is involved in many metabolic, nutritional, physiological and immunological interactions with the host [1].

The host immune system plays an important role in distinguishing between commensal and pathogenic bacteria. On one hand, the immune system needs to stay alert to recognize potential pathogens, and on the other hand, it has to tolerate the commensal bacteria inhabiting the gut [2]. This homeostasis is achieved through pattern recognition receptor (PRR) families expressed in immune cells. PRRs, such as Toll-like receptors (TLRs) and nucleotide binding and oligomerization domain-like receptors (NLRs), identify microbe-associated molecular patterns (MAMPs). MAMPs are molecules associated with both commensal and pathogenic microorganisms. Another important component of the mucosal immune system are the secretory immunoglobulins, such as IgA and IgG, which are secreted by plasma cells and function by excluding bacteria from the epithelium [3, 4]. Defining the immune-modulatory capacity of members of the microbiota is essential in understanding their involvement in the establishment of mucosal tolerance and balanced intestinal immune responses. There is also growing evidence about the influence of the gut microbiota on the systemic immune system, and consequently, the development of autoimmune diseases [5].

One of the key players in the colonic mucus-associated microbiota is *Akkermansia muciniphila*, which colonizes a considerable part of the human population and comprises 1–4% of the fecal microbiota of healthy adults [6, 7]. This bacterium is highly adapted to its living environment as it is capable of using mucin as the sole carbon and nitrogen source. Levels of *A*. *muciniphila* have been shown to be inversely correlated with several disorders [8], such as inflammatory bowel diseases (IBD) [9, 10], appendicitis [11], obesity [12, 13] and diabetes [14], but not much is known about its immunological mechanism of action.

The impact of *A*. *muciniphila* on the host has been studied in (mono-associated) mice and organoids, where most of the genes affected by the bacteria were implicated in immune and metabolic responses [13, 15, 16]. The induction of immune response-associated genes was most obvious in the colon of *A*. *muciniphila* colonized mice, where over 60 genes, including 16 genes encoding cluster of differentiation (CD) antigen markers and 10 genes encoding immune cell membrane receptors were up-regulated upon exposure. The impact on host metabolism is in line with the fact that *A*. *muciniphila* can have an inhibiting effect on obesity and diabetes development. The abundance of *A*. *muciniphila* decreased in obese and type 2 diabetic mice, and treatment with the bacteria reversed high-fat diet induced metabolic disorders, such as adipose tissue inflammation [17]. This was confirmed in a later study where *A*. *muciniphila*-administered high-fat diet fed mice showed improved glucose tolerance and an increase in the number of goblet cells and adipose tissue-resident CD4+ Foxp3+ regulatory T cells [18]. Conversely to these studies implying a protective effect of *A*. *muciniphila* on intestinal barrier function and immune stimulation, several mouse studies have reported increased numbers of these mucosal bacteria in dextran sodium sulfate (DSS)-induced colitis [19–21]. This could be explained by a simple outgrowth of *A*. *muciniphila* in response to the thickening of the mucus layer during DSS-induced colitis. A similar explanation can rationalize the observation that *A*. *muciniphila* administration in a minimal community appeared to aggravate *Salmonella enterica* Typhimurium-induced gut inflammation in a gnotobiotic mouse model [22].

The aim of this study was to characterize the immune-modulatory properties of *A*. *muciniphila* MucT by measuring cytokine production in human derived peripheral blood mononuclear cells (PBMCs) and activation of inflammatory pathways on reporter cell lines expressing either TLR2/4/5/9 or NOD2-receptor. The immune response of *A*. *muciniphila* was compared to two other commensals, *Faecalibacterium prausnitzii* A2-165 and *Lactobacillus plantarum* WCFS1. A proteomics approach was used to identify candidate-signaling molecules from bacterial fractions, and a collection of these proteins was purified from overproducing *Escherichia coli* clones. These proteins were tested for their capacity to induce TLR2-signaling, cytokine production and to affect trans-epithelial resistance (TEER) in Caco-2 model system. Localization of specific proteins was studied with immunofluorescence labeling using specific antibodies.

## Materials and methods

### Bacterial growth conditions

*Akkermansia muciniphila* MucT (ATTC BAA-835) was grown in a basal medium as described previously [7]. The medium was supplemented with either hog gastric mucin (0.5%, Type III; Sigma-Aldrich, St. Louis, MO, USA), a mix of sugars (D-glucose, L-fucose, N-acetylglucosamine, N-acetylgalactosamine; 2.5 mM each, Sigma-Aldrich) or glucose (10 mM, Sigma-Aldrich). The medium without mucin was supplemented with tryptone (8 g/l, Oxoid Ltd, Basingstoke, Hampshire, England) and L-threonine (2 mM, Sigma-Aldrich). Incubations were performed in serum bottles sealed with butyl-rubber stoppers at 37°C under anaerobic conditions provided by a gas phase of 182 kPa (1.5 atm) N_2_/CO_2_ (80/20 ratio). Growth was measured by spectrophotometer as optical density at 600 nm (OD600).

*Faecalibacterium prausnitzii* A2-165 was grown anaerobically at 37°C in YCFA medium supplemented with 33 mM glucose [23]. *Lactobacillus plantarum* WCFS1 was grown aerobically and *Bifidobacterium breve* DSM-20213 anaerobically at 37°C in Difco™ Lactobacilli MRS broth (Becton Dickinson, Sparks, USA).

### Peripheral blood mononuclear cells assay

Peripheral blood of three healthy donors was received from the Sanquin Blood Bank, Nijmegen, The Netherlands. Peripheral blood mononuclear cells (PBMCs) were separated from the blood of healthy donors using Ficoll-Paque Plus gradient centrifugation according to the manufacturer's protocol (Amersham biosciences, Uppsala, Sweden). After centrifugation the mononuclear cells were collected, washed in Iscove's Modified Dulbecco's Medium (IMDM) + Glutamax (Invitrogen, Breda, The Netherlands) and adjusted to 0.5 × 10^6^ cells/ml in IMDM + Glutamax supplemented with penicillin (100 U/ml) (Invitrogen), streptomycin (100 μg/ml) (Invitrogen), and 10% heat inactivated Fetal Bovine Serum (FBS, Lonza, Basel, Switzerland). PBMCs (0.5 × 10^6^ cells/well) were seeded in 48-well tissue culture plates.

Per donor a negative control (just medium), and a positive control (LPS from *E*. *coli* 1 μg/ml) were used. PBMCs were stimulated with live bacteria or bacterial fractions. For the heat-killed cells the bacterial culture was kept at 99°C for 10 min. The ratio of PBMC to bacteria was 1:10. Cells were incubated for 24 hours and the culture supernatants were collected for cytokine analysis. Cytokine levels of IL-6, IL-8, IL-10, TNF-α, IL-1β and IL-12p70 were measured using multiplex analysis (Human inflammation CBA kit, Becton Dickinson) according to the manufacturer’s protocol on a FACS CantoII (Becton Dickinson) and analyzed using BD FCAP software (Becton Dickinson). The detection limits according to the manufacturer were as follows: 3.6 pg/ml IL-8, 7.2 pg/ml IL-1β, 2.5 pg/ml IL-6, 3.3 pg/ml IL-10, 3.7 pg/ml TNF-α, 1.9 pg/ml IL-12p70.

### *In vitro* culture and stimulation of human HEK-Blue hTLR2/4/5/9/NOD2 cell lines

**Fig 1:**
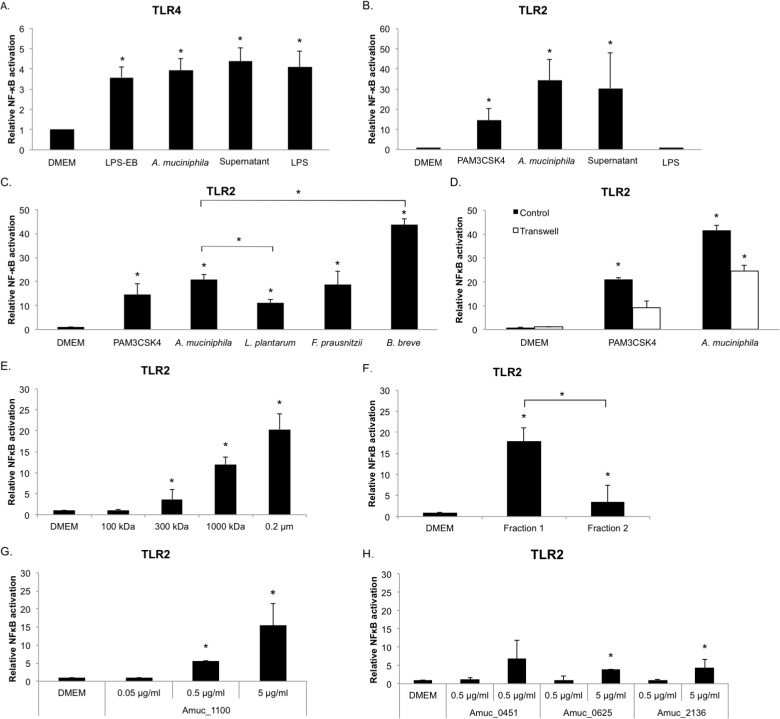
*A*. *muciniphila* activates signaling pathways through TLR2 and TLR4. (A) TLR4 signaling by live *A*. *muciniphila* (~10^7^ bacteria/well), *A*. *muciniphila* supernatant (~5 μg of protein/well) and LPS isolated from *A*. *muciniphila* (concentration corresponds to amount of LPS in ~10^7^ *A*. *muciniphila* cells, ~10^5^–10^6^ EU/ml). DMEM: medium control, LPS-EB: positive control (concentration corresponds to amount of LPS in ~10^7^ *E*. *coli* cells). (B) TLR2 signaling by live *A*. *muciniphila* (~10^7^ bacteria/well), *A*. *muciniphila* supernatant (~5 μg of protein/well) and LPS. DMEM; medium control, PAM3CSK4; positive control. (C) TLR2 signaling by live *A*. *muciniphila*, *L*. *plantarum*, *F*. *prausnitzii* and *B*. *breve* (~10^6^ bacteria/well). (D) TLR2 signaling in a Transwell system compared to control (i.e. samples not separated from the cell line by a membrane, ~4 × 10^7^ bacteria/well). (E) TLR2 signaling by filtrated supernatant signaling molecules. (F) TLR2 signaling by *A*. *muciniphila* bacterial fractions (1 μg of protein/well). (G) TLR2 signaling by *A*. *muciniphila* purified protein Amuc_1100 (0.01, 0.1 and 1 μg of protein/well). (H) TLR2 signaling by *A*. *muciniphila* purified proteins Amuc_0451, Amuc_0625 and Amuc_2136 (0.1 and 1 μg of protein/well). DMEM; medium control, *, P<0.05 compared to DMEM. All experiments were performed in triplicate and statistical analysis was performed by one-way analysis of variance (ANOVA) followed by Tukey’s HSD if homogeneity of variance was met or Games-Howell if variance was unequal.

For the inflammatory pathway analysis HEK-Blue hTLR2, hTLR4, hTLR5, hTLR9 and hNOD2 cell lines (Invivogen, CA, USA) were used. The cell lines were obtained directly from Invivogen where they were authenticated. The cell lines were regularly tested for contamination by *Mycoplasma* using a PCR-based method. Stimulation of the receptors with the corresponding ligands activates NF-κB and AP-1, which induces the production of secreted embryonic alkaline phosphatase (SEAP), the levels of which were measured by spectrophotometer (Spectramax). All cell lines were grown and subcultured up to 70–80% of confluency using as a maintenance medium Dulbecco's Modified Eagle Medium (DMEM) supplemented with 4.5 g/l D-glucose, 50 U/ml penicillin, 50 μg/ml streptomycin, 100 μg/ml Normocin, 2 mM L-glutamine, and 10% (v/v) of heat-inactivated FBS. For each cell line, an immune response experiment was carried out by seeding HEK-blue cells (volume 180 μl, cell amounts listed in Table B in S1 File) in flat-bottom 96-well plates and stimulating them by addition of 20 μl bacterial suspensions. The concentrations of the suspensions are indicated in Fig 1. The 96-well plates were incubated for 20–24 h at 37°C in a 5% CO2 incubator. Receptor ligands Pam3CSK4 (10 ng/ml for hTLR2), LPS-EB (50 ng/ml for hTLR4), RecFLA-ST (0.1 ng/ml for hTLR5), ODN 2006 (50 μM for hTLR9) and L18-MDP (0.1 ng/ml for hNOD2) were used as positive control whereas maintenance medium without any selective antibiotics was used as negative control. SEAP secretion was detected by measuring the OD600 at 15 min, 1 h, 2 h, and 3 h after addition of 180 μL of QUANTI-Blue (Invivogen) to 20 μL of induced HEK-Blue hTLR2/4/5/9/NOD2 supernatant. The data shown here are from the 1 h measurement point. Experiments were performed in triplicate.

### Transwell assay

To study the TLR2 signaling activity of secreted molecules, the bacteria were separated from the cell line using Transwell (Corning, USA) cell culture membrane inserts (0.4 μm pore size). Bacterial suspension (100 μl, ~4 × 10^7^ bacteria/well) was added either directly to wells containing HEK-TLR2 cells or first to the Transwell insert compartment, which was then inserted into the wells with HEK-TLR2 cells (volume 900 μl). The plates were incubated and SEAP secretion measured as described above.

### Bacterial filtrates

To study the size of the signaling molecules, the bacterial supernatant was filtered using filters of different pore sizes and molecular mass cut off sizes. The following filters were used: 0.45 μm and 0.2 μm polyethersulfone syringe filters (Advanced Microdevices, Ambala Cantt., India), 1000 kDa Vivaspin 20 Polyethersulfone ultrafiltration unit (Sartorius, Goettingen, Germany), 3K and 300K Pall Nanosep® centrifugal device with Omega membrane (Pall corporation, Ann Arbor, Michigan), 10K Amicon® Ultra regenerated cellulose centrifugal filter (Merck Millipore Ltd., Cork, Ireland), Vivaspin 500 with 30,000 MWCO (Polyethersulfone) Membrane Concentrator (Vivascience, Sartorius Group, Hannover, Germany). 500 μl of supernatant was passed through the filter and the filtrate was used in the assays.

### Bacterial fractionation method

The membranes of *A*. *muciniphila* were isolated from cultures grown with glucose as the carbon source with sucrose density-gradient centrifugation, as described previously [24]. The samples were stored in 2 ml low binding tubes (Eppendorf, Hamburg, Germany) at −20°C. Qubit® Protein Assay Kit (Life technologies, Oregon, USA) was used according to the manufacturer's instructions to determine the protein content of cell extracts. Samples were loaded on a 10% acrylamide separation gel (25201, Precise™ Protein Gels, Thermo Scientific, Rockford, IL, USA) using the mini-PROTEAN 3 cell (Bio-Rad Laboratories, Hercules, CA, USA). The electrophoresis procedure was according to the manufacturer's instructions. Gels were stained using CBB R-250 as indicated in the protocol of the mini-PROTEAN 3 system.

In-gel digestion of proteins and purification of peptides were done following a modified version of a protocol described previously [25]. Disulfide bridges in proteins were reduced by covering whole gels with reducing solution (10 mM dithiothreitol, pH 7.6, in 50 mM NH_4_HCO_3_), and the gels were incubated at 60°C for 1 h. Alkylation was performed for 1 h by adding 25 mL of iodoacetamide solution (10 mM iodoacetamide in 100 mM Tris-HCl, pH 8.0). Gels were thoroughly rinsed with dd H_2_O water in between steps. Each lane of SDS-PAGE gels was cut into one slice, and the slices were cut into approximately 1 mm^3^ cubes and transferred to separate 0.5 ml protein LoBind tubes (Eppendorf). Enzymatic digestion was done by adding 50 μl of trypsin solution (5 ng/μl trypsin in 50 mM NH_4_HCO_3_) to each tube, and by incubating at room temperature overnight with gentle shaking. Extraction of peptides was performed with manual sonication in an ultrasonic water bath for 1 s before the supernatant was transferred to a clean protein LoBind tube. Trifluoroacetic acid (10%) was added to the supernatant to reach a pH between 2 and 4. The supernatant was used for LC-MS/MS analysis. Samples were measured by nLC–MS/MS with a Proxeon EASY nLC and a LTQ-Orbitrap XL mass spectrometer as previously described [26].

LC–MS data analysis was performed as described previously [25], with false discovery rates (FDRs) set to 0.01 on peptide and protein level, and additional result filtering (minimally 2 peptides necessary for protein identification of which at least one is unique and at least one is unmodified). To analyze the abundance of proteins in the fractions, their label-free quantification (LFQ) intensities were compared [27]. Non-existing LFQ intensity values due to not enough quantified peptides were substituted with a value lower than the LFQ intensity value for the least abundant, detected peptide.

### Plasmid constructs and protein production

The genes Amuc_0451, Amuc_0625, Amuc_1100, and Amuc_2136 were amplified by PCR without their signal sequence, with primers as specified in Table A in S1 File. PCR products of the genes were cloned into either pET-24d or pET-26b vectors (Novagen®, Merck Millipore, MA, USA). For genes Amuc_0451, Amuc_0625, and Amuc_1100 pET-26b was used with restriction sites NdeI and XhoI. For Amuc_2136 gene, pET-24d was used and the PCR product containing PciI and XhoI restriction sites was cloned at the NcoI and XhoI sites of the vector.

*E*. *coli* XL1Blue or TOP10 cells were transformed with constructed plasmids by electroporation or heat shock, respectively. Cells with kanamycin resistance were selected by plating the transformed cells on LB agar plates containing 50 μg/ml kanamycin. Plasmids isolated from colonies on these plates were checked for having the right insert length by PCR and subsequently, isolated plasmids were sequenced to confirm the right insert.

*E*. *coli* BL21(DE3) cells were transformed with the right plasmid for protein expression. LB broth containing kanamycin (50 μg/ml) was inoculated with overnight culture and grown with shaking at 220 rpm at 37°C until exponential phase, and protein production was induced by adding IPTG up to 1 mM. After three hours of induction, cells were pelleted by centrifuging 10 min at 5000 g and cell pellets stored at –20°C until lysis.

Cell pellets were resuspended and lysed using lysozyme and sonification (Sonifier 450, Branson Ultrasonics Corporation, Danbury, CT). Supernatant was collected after centrifugation and proteins were His-tag purified by metal affinity purification under native conditions using Ni-NTA His•Bind Resin (Novagen®, Merck Millipore, MA, USA). Elution buffer was exchanged for a 50 mM Tris-HCl, 50 mM NaCl, pH of 7.4 buffer by using 5 ml resin bed Zeba spin columns (Pierce, Rockford, IL, USA). After buffer exchange, protein content was measured by BCA assay (Pierce) and proteins were stored at -20°C.

### Immunofluorescence microscopy

Rabbit antibodies were raised against the purified recombinant Amuc_1100 protein and these were used in immunomicroscopic analysis of its location in *A*. *muciniphila*. Total antibodies raised against *A*. *muciniphila* whole cells were used as a control. The immunization was done in Eurogentec (Seraing, Belgium) and the Laboratory Animal Centre of University of Helsinki (Finland) as described previously [28].

Immunofluorescence staining was used to confirm the presence of Amuc_1100 on the surface of *A*. *muciniphila* as described previously [29]. Briefly, *A*. *muciniphila* cells were cultivated for 24 h with glucose as the carbon source, washed with phosphate-buffered saline (PBS), and fixed with 3.5% (w/v) paraformaldehyde in PBS prior to labeling with *A*. *muciniphila* whole cell antiserum or anti-Amuc_1100 pre-immune serum as the primary antiserum and Alexa-488 (Invitrogen)-conjugated anti-rabbit IgG (1 μg/ml) as the secondary antibody. Bacteria were then examined with an epifluorescence microscope (Leica DM 4000B) equipped with a filter for the Alexa-488 label (excitation, 450 to 490 nm; emission, 515 nm), and images were digitally recorded using CellP imaging software for life sciences microscopy (Soft Imaging System GmbH).

### Extraction of LPS

*A*. *muciniphila* LPS was extracted using the hot phenol-water extraction method as described previously [30], with minor modifications. Briefly, bacterial cells from 5 ml overnight cultures were collected by centrifugation, washed once with water and resuspended into 500 μl of ultrapure water. The bacterial suspensions were warmed up at 65°C and then mixed with an equal volume of water-saturated phenol preheated to 65°C. The mixture was incubated at 65°C for 10 min and then transferred to ice to cool down. After centrifugation at 4°C for 5 min, the aqueous layer was carefully transferred to a new Eppendorf tube and the incubation with an equal volume of hot phenol was repeated twice. After this two volumes of acetone were added to the aqueous layer to precipitate LPS. The suspension was incubated at -20°C for two hours after which it was centrifuged at 4°C for 10 minutes and the pellet was dissolved in 50 μl LPS-free water. The quality of the LPS was checked by silver staining (Fig B in S1 File) and quantity of the LPS was checked by EndoLISA® Endotoxin Detection Assay (Hyglos GmbH, Bernried am Starnberger See, Germany) according to manufacturer’s protocol.

### Transepithelial Electrical Resistance (TEER) assay

Caco-2 cells (5 x 10^4^ cells/insert) were seeded in Millicell cell culture inserts (3 μm pore size; Merck Millipore) and grown for 8 days. The growth conditions of Caco-2 cells were as described previously [31]. Bacterial cells were washed once with RPMI 1640, and applied onto the inserts at OD600 of 0.25 in RPMI 1640. Purified *A*. *muciniphila* proteins Amuc_0451, Amuc_0625, Amuc_1100, and Amuc_2136 were applied onto the inserts at concentrations of 0.05, 0.5 and 5 μg/ml. The transepithelial resistance was determined with a Millicell ERS-2 TEER meter (Merck Millipore) from cell cultures at time points 0 h, 24 h, and 48 h after addition of bacterial cells or proteins.

### Statistical analysis

Data are expressed as means ± standard deviation. Statistical analysis of the results from the HEK-Blue cell lines and cytokine analysis was performed by one-way analysis of variance (ANOVA) followed by Tukey’s HSD if homogeneity of variance was met or Games-Howell if variance was unequal. IBM SPSS software (IBM SPSS Statistics 22) was used for analysis; p values < 0.05 were considered significant.

## Results

### *A*. *muciniphila* stimulates species-specific cytokine patterns when compared to other commensals

Stimulation of peripheral blood mononuclear cells (PBMCs) with *A*. *muciniphila* MucT resulted in induction of both anti- and pro-inflammatory cytokines (IL-1β, IL-6, IL-8, IL-10 and TNF-α). This induction was seen for live cells as well as for heat-killed cells and supernatant (Table 1). Among the measured cytokines, IL-10, IL-8, IL-6 and TNF-α were the highest induced.

**Table 1:** Effect of bacteria and bacterial fractions on cytokine production of human PBMCs.

**Cytokine**	**Live**		**Heat-killed**	**Supernatant**	**Live**		**Live**
(pg/ml)	***A*. *muciniphila***		***A*. *muciniphila***	***A*. *muciniphila***	***F*. *prausnitzii***		***L*. *plantarum***
IL-1β	894 ± 298		392 ± 71	1650 ± 510	870 ± 301		894 ± 298
IL-6	18029 ± 309		13477 ± 2014	23225 ± 8102	10178 ± 2648		7028 ± 2812
IL-8	60018 ± 18229		54230 ± 9030	86171 ± 32298	48354 ± 10526		33085 ± 16760
IL-10	823 ± 310		638 ± 118	1221 ± 310	834 ± 388		333 ± 215
TNF-α	1920 ± 349		957 ± 568	2095 ± 1249	5545 ± 2615		5459 ± 2830
IL-12p70	< 2		< 2	< 2	< 2		253 ± 293
**Supernatant filtrates**							
Cytokine (pg/ml)		**10 kDa filtrate**	**30 kDa filtrate**	**100 kDa filtrate**	**300 kDa filtrate**	**1000 kDa filtrate**	
IL-1β		< 8	< 8	62 ± 29	678 ± 350	949 ± 522	
IL-6		22 ± 23	569 ± 430	2556 ± 1049	18697 ± 7097	18717 ± 7326	
IL-8		4321 ± 4530	11928 ± 7128	29040 ± 14973	74208 ± 28348	73695 ± 36442	
IL-10		< 4	6 ± 4	43 ± 28	937 ± 287	1215 ± 379	
TNF-α		< 4	37 ± 48	247 ± 255	1476 ± 849	1188 ± 930	
IL-12p70		< 2	< 2	< 2	< 2	< 2	
**Sucrose density-gradient separated fractions and purified Amuc_1100**					**Controls**		
Cytokine		**Fraction 1**	**Fraction 2**	**Amuc_1100**	**Medium**		**LPS**
(pg/ml)		(4.5 μg/ml)	(4.5 μg/ml)	(4.5 μg/ml)			(1 μg/ml)
IL-1β		437 ± 225	< 8	504 ± 227	< 8		324 ± 121
IL-6		9312 ± 3329	414 ± 364	12508 ± 2362	< 4		13299 ± 5460
IL-8		64877 ± 19528	13339 ± 5039	45432 ± 12507	489 ± 291		60063 ± 5765
IL-10		941 ± 404	21 ± 18	526 ± 180	< 4		319 ± 157
TNF-α		2165 ± 883	60 ± 35	1317 ± 885	< 4		1392 ± 622
IL-12p70		< 2	< 2	< 2	< 2		< 2

Data are presented as mean ± SD, n = 3 donors. Per donor a negative control (just medium), and a positive control (LPS from *E*. *coli* 1 μg/ml) were used.

**Fig 2:**
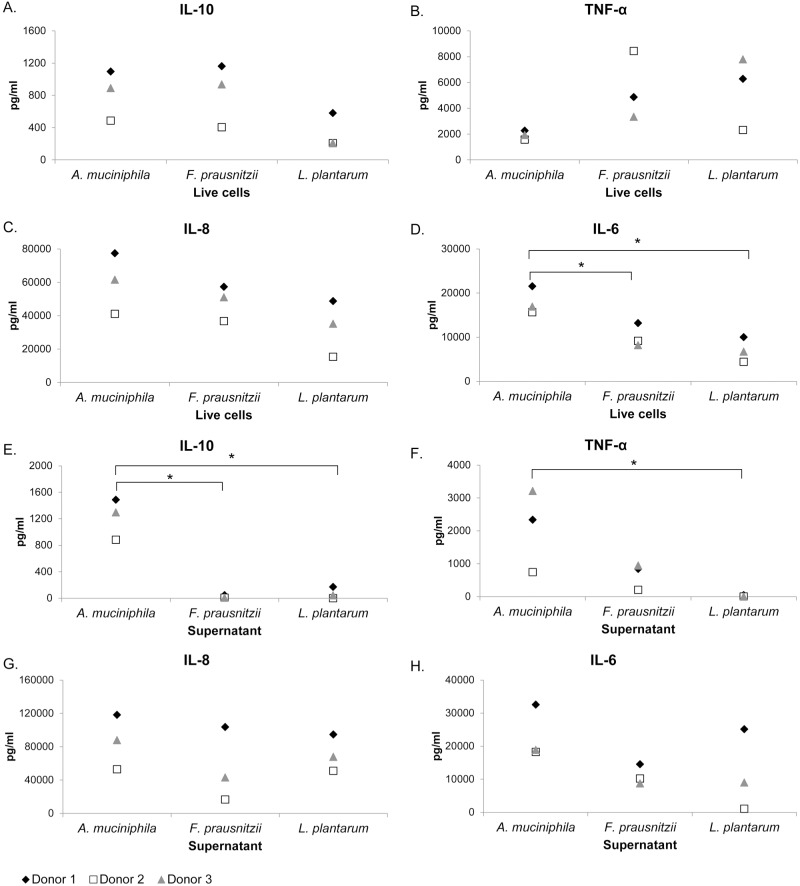
Effect of *A*. *muciniphila*, *F*. *prausnitzii* and *L*. *plantarum* on cytokine production of human PBMCs. IL-10 (A), TNF-α (B), IL-8 (C) and IL-6 (D) responses of human PBMCs (n = 3 donors) stimulated with *A*. *muciniphila*, *F*. *prausnitzii* and *L*. *plantarum* live cells. IL-10 (E), TNF-α (F), IL-8 (G) and IL-6 (H) responses of human PBMCs (n = 3 donors) stimulated with *A*. *muciniphila*, *F*. *prausnitzii* and *L*. *plantarum* supernatant. *, P<0.05. Statistical analysis was performed by one-way analysis of variance (ANOVA) followed by Tukey’s HSD if homogeneity of variance was met or Games-Howell if variance was unequal.

*A*. *muciniphila* immune stimulation in PBMCs was compared to two other established beneficial gut microbes, *F*. *prausnitzii* A2-165 and *L*. *plantarum* WCFS1. The stimulation of PBMCs led to a microbe-specific pattern of all tested microorganisms. In comparison to *F*. *prausnitzii*, *A*. *muciniphila* induced higher levels of IL-6 and IL-8, while levels of IL-10 were similar and levels of TNF-α were lower (Table 1, Fig 2A–2D). Compared to *L*. *plantarum*, *A*. *muciniphila* induced higher levels of IL-6, IL-8 and IL-10 but less TNF-α (Table 1, Fig 2A–2D). PBMCs were also stimulated with the supernatants of these bacteria leading to a markedly different cytokine response for IL-10 and TNF-α, whereas IL-8 and IL-6 showed more similar patterns compared to live bacteria (Fig 2E–2H). Remarkably, *A*. *muciniphila* supernatant induced significantly more IL-10 than *F*. *prausnitzii* supernatant and more IL-10 and TNF-α than *L*. *plantarum* supernatant (Fig 2E and 2F). When analysing the TNF-α/IL-10 ratio as a measure of inflammation it was found that *A*. *muciniphila* had a low inflammatory potential in comparison to *F*. *prausnitzii and L*. *plantarum* (Fig A in S1 File).

### *A*. *muciniphila* activates the NF-κB pathway through TLR4 and TLR2 receptors

To determine which intestinal receptors are involved in immune stimulation of *A*. *muciniphila*, reporter cell lines expressing TLR2, TLR4, TLR5, TLR9 or NOD2 receptors were employed. The strongest activation was seen on TLR4 (Fig 1A) and TLR2 (Fig 1B). *A*. *muciniphila* did not activate TLR5 and TLR9, and only minor activation was seen for NOD2 [32].

TLR2 responses were higher in the presence of *A*. *muciniphila* compared to *L*. *plantarum* (Fig 1C), but there was no significant difference between the TLR2 response induced by *F*. *prausnitzii* and *A*. *muciniphila* (Fig 1C). However, the TLR2 response towards *A*. *muciniphila* was lower than the induction by the Gram-positive *Bifidobacterium breve* DSM-20213 (Fig 1C).

TLR4 is an important receptor for recognizing Gram-negative sensitive lipopolysaccharide (LPS). We first verified the presence of LPS in *A*. *muciniphila* by using a protocol to extract LPS from bacterial cells and applying silver staining to visualize it on a gel (Fig B in S1 File). In these reporter cell line-experiments both live bacteria and *A*. *muciniphila* LPS significantly stimulated NF-κB dependent secreted embryonic alkaline phosphatase (SEAP) production via TLR4 (Fig 1A). On top of this, *A*. *muciniphila* LPS induced production of IL-8, IL-6 and low amounts of IL-10 and TNF-α in PBMCs. As expected, *A*. *muciniphila* LPS did not induce a TLR2 response (Fig 1B).

### 30 kDa outer membrane pili-like protein (Amuc_1100) is a strong TLR2 activator and induces cytokines in PBMCs

*A*. *muciniphila* supernatant activated TLR2 as the NF-κB activity persisted while bacteria were separated from the cell line by a membrane in a Transwell assay (Fig 1D). This indicates *A*. *muciniphila* can activate TLR2 with both cell derived fragments and extracellular molecules. The main fermentation products in the supernatant of *A*. *muciniphila* are acetate and propionate, but no effect on NF-κB activity was observed for these fatty acids at a concentration of 1 mM (Fig C in S1 File). Using centrifugal membrane filters of differing pore sizes, we could demonstrate that for TLR2 and cytokine induction the size of signaling molecules from the supernatants had to be larger than 100 kDa (Fig 1E). The production of IL-8, IL-1β, IL-6, IL-10 and TNF-α by PBMCs increased along with the molecule size of the filtrated supernatant (Table 1).

**Fig 3:**
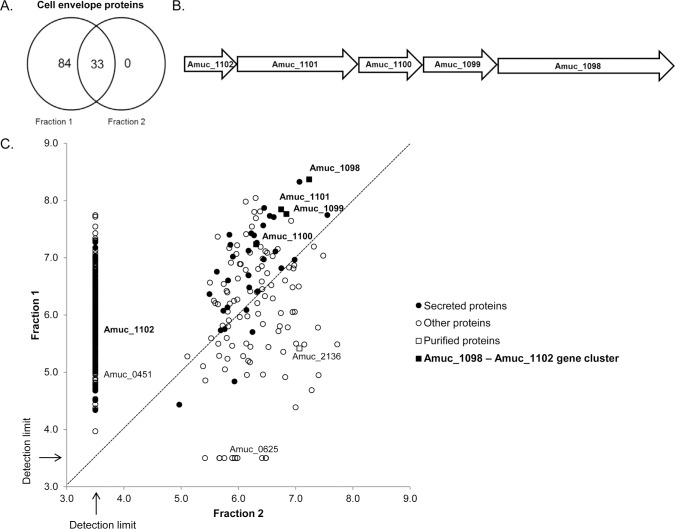
Proteins encoded by the gene cluster Amuc_1098 to Amuc_1102 are found abundantly in a fraction enriched for membrane and cell-envelope proteins. (A) Number of *A*. *muciniphila* membrane and cell-envelope proteins detected with LC-MS/MS in two different fractions from a sucrose density-gradient separation method. (B) Amuc_1098 to Amuc_1102 gene cluster. (C) Abundance of proteins found in Fraction 1 vs. Fraction 2. Relative abundances of the proteins are presented on a log10 scale. A log10 relative abundance of 3.5 represents proteins that were not detected or were under the detection limit.

Next we separated bacterial membranes from intracellular proteins with sucrose density-gradient centrifugation. LC-MS/MS-analysis was done on four different fractions along the gradient to identify the proteins and determine their relative amounts. Two samples (Fraction 1 & 2) separated with sucrose density-gradient centrifugation were found to differ notably in the relative amount of cell envelope proteins. In total 117 cell envelope proteins were detected in these fractions, with 84 of them exclusively found in Fraction 1 (Fig 3A). Fraction 1 was especially enriched for proteins involved in protein transport and secretion, in comparison to Fraction 2. Fraction 2 had a more heterogeneous protein content based on protein function, but overall lower diversity of proteins. Among the most abundant proteins in Fraction 2 were mucin-degrading enzymes (glycosyl hydrolase, beta-galactosidase, N-acetylhexosaminidase) and other enzymes (alanine-glyoxylate transaminase, hyalurononglucosaminidase). These fractions were also tested on the TLR2 cell line and PBMCs in equal protein concentrations. Fraction 1 induced higher TLR2 activity than Fraction 2 (Fig 1F). Fraction 1 also induced higher cytokine production in PBMCs as compared to Fraction 2 (Table 1).

**Fig 4:**
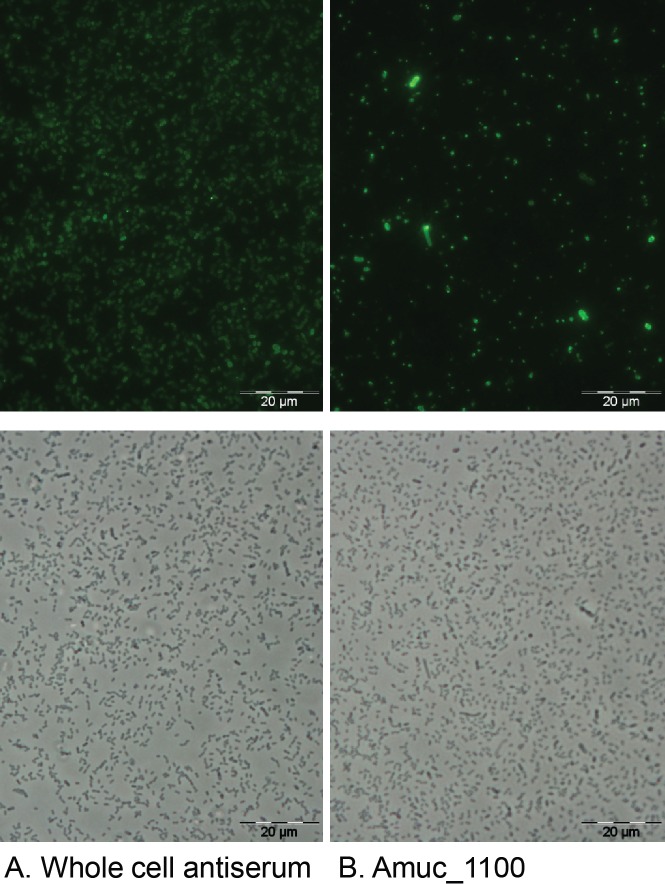
Amuc_1100 is located on the outer membrane of *A*. *muciniphila*. Immunofluorescence staining of *A*. *muciniphila* cells with whole cell antiserum (A) or anti-Amuc_1100 (B) and Alexa-488-conjugated secondary IgG. Phase-contrast images of the same microscopic fields are shown below. Pictures were cropped from the original image.

Based on proteomics analysis with LC–MS/MS we discovered that the relative abundance of all proteins that were encoded by a gene cluster corresponding to locus tags Amuc_1098 to Amuc_1102 (Fig 3B), was at least ten times higher in Fraction 1 compared to Fraction 2 (Fig 3C). Previously, we have shown that proteins from this gene cluster are highly abundant also in the whole proteome of *A*. *muciniphila*, with Amuc_1098 being the most abundant outer membrane protein [24]. Amuc_1098 is predicted to encode a type II and type III secretion system protein and Amuc_1101 is predicted to encode a cell division protein FtsA. The other three genes (Amuc_1099, Amuc_1100, Amuc_1101) are annotated as hypothetical proteins. All the genes, except for Amuc_1101, have a signal sequence at the N terminus, indicating they are destined towards the secretory pathway and may be involved in forming the pili-like structures as described by Derrien et al., 2004 [7]. Recently, administration of the purified recombinant Amuc_1100 protein was shown to improve glucose tolerance and induce a lower body weight and fat mass gain in mice fed a high-fat diet in comparison to untreated mice on the same diet [32]. We used immunofluorescence labeling to localize the proteins in the bacteria, and successfully identified Amuc_1100 (32 kDa) as an outer membrane protein (Fig 4B). However, some variation was observed in the intensity of the labeling of the *A*. *muciniphila* cells with the anti-Amuc_1100 antibodies (Fig 4B). Similarly, the labeling intensity also varied, but in a less extensive way, with the whole cell antibody (Fig 4A).

Purified recombinant Amuc_1100 protein gave specific induction of TLR2 (Fig 1G) and was able to induce IL-1β, IL-6, IL-8, IL-10 and TNF-α production in PBMCs (Table 1). As the genes Amuc_1101 and Amuc_1102 did not lead to overproduced soluble proteins, we were not able to test the effect of these proteins on the immune response. As a control to Amuc_1100, secreted enzymes that are involved in *A*. *muciniphila* mucin degradation were tested. It was found that the enzymes Amuc_0451 (sulfatase), Amuc_0625 (exo-alpha-sialidase), and Amuc_2136 (glycoside hydrolase) are abundantly produced by *A*. *muciniphila* and dose-dependent TLR2-signaling was detected for each of them (Fig 1H). However, the TLR2-signaling response tended to be lower for these periplasmic enzymes in comparison to Amuc_1100.

**Fig 5:**
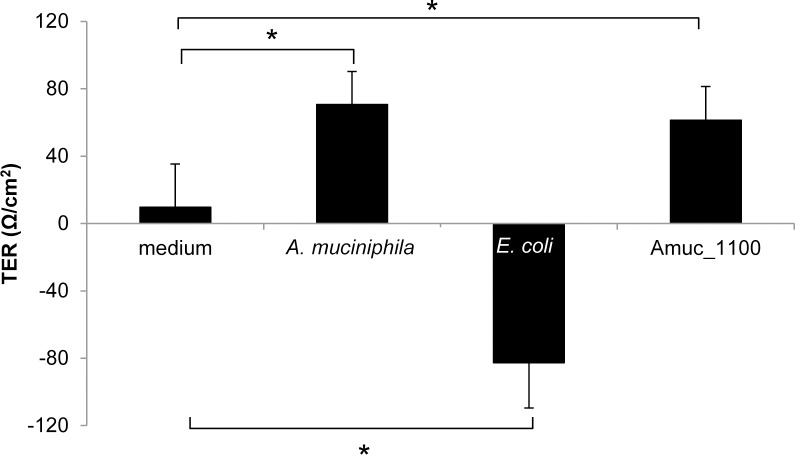
*A*. *muciniphila* and outer membrane protein Amuc_1100 increase the development of transepithelial electrical resistance. The impact of *A*. *muciniphila*, purified protein Amuc_1100 (0.05 μg/ml) or *E*. *coli* on the TEER development of Caco-2 monolayer after 24 h of stimulation. Mean and standard deviations from three parallel wells are shown. Significant differences (p < 0.05) in the TEER values as compared to control (growth medium without bacteria) at 24 h are indicated with an asterix. Statistical analysis was performed by one-way analysis of variance (ANOVA).

Additionally, we tested the impact of *A*. *muciniphila* and the purified recombinant proteins on the development of the integrity of epithelial cell layer by determining the TER of Caco-2 monolayers. *A*. *muciniphila* showed a significantly increased TEER after 24 h of co-cultivation with the Caco-2 cells as reported previously [33] (Fig 5). In our assay also Amuc_1100 showed a significantly increased TEER at 24 h (Fig 5), whereas the other purified proteins (Amuc_2136, Amuc_0625 and Amuc_0451) did not significantly increase TEER (Fig D in S1 File). *E*. *coli* is known to have adverse effects on epithelial cell monolayer integrity and decreased the TEER (Fig 5) [34].

## Discussion

*A*. *muciniphila* MucT was capable of inducing a wide range of immune-modulatory responses *in vitro*. The immune modulatory capacity was traced back to large molecule complexes with a molecular size of over 100 kDa. Subsequently, we showed that a bacterial fraction enriched in cell envelope proteins, presumably including large structures exposed to the surface of the bacteria, induced high TLR2 signaling and cytokine production in PBMCs. We showed this fraction to be highly enriched in a set of proteins encoded by the gene cluster Amuc_1098—Amuc_1102 that could constitute the pili-like structures observed in electron microscope images [7]. Amuc_1100 is part of this gene cluster, and its 30-kDa product could be overproduced in *E*. *coli* and found to induce production of IL-6, IL-8 and IL-10 in PBMCs. Moreover, using immunofluorescence microscopy we could localize the Amuc_1100 protein at the outside of *A*. *muciniphila* cells, compatible with its location in pili-like structures. Some heterogeneity in the immunolabeling was observed, possibly reflecting different amounts of pili per cell. As *A*. *muciniphila* is located in the mucus layer, not far from the epithelial cells, it may benefit from these types of appendages when interacting with the host.

In addition, a few secreted mucin-degrading enzymes also activated PBMCs and TLR2. Previously it was reported that *A*. *muciniphila* extracellular vesicles (EV) stimulate IL-6 secretion, but pre-treatment of a colon epithelial cell line with these EV before treating them with *E*. *coli* EV, decreased IL-6 production as compared to treatment with *E*. *coli* EV alone [20]. It is unclear which proteins are present in the *A*. *muciniphila* EV that trigger the response, and whether mucin fragments from the culture medium may have confounded the results. In our study *A*. *muciniphila* was grown on non-mucus medium for all the immune assays to prevent compounds of hog gastric mucin from interfering with the immune response.

IL-8, IL-6, IL-1β, IL-10 and TNF-α were induced by *A*. *muciniphila* in human derived PBMCs, indicating it cannot be strictly defined as anti- or pro- inflammatory, but may instead have a more complex role in preserving the balance of the gut ecosystem. Interestingly, live bacteria induced significantly higher IL-1β production than heat-killed bacteria, suggesting that cell derived fragments as well as secreted compounds have an important role in the immune signaling. The immunomodulatory outer membrane structures may have also been damaged during the heat treatment. Stimulation of PBMCs with *A*. *muciniphila* led to production of proinflammatory IL-8. However, a recent study showed that *A*. *muciniphila* induced IL-8 production in enterocytes at 100-fold higher cell concentrations as compared to *E*. *coli*, suggesting a fairly low inflammatory potential in the gut [33].

The immune modulatory effects observed for *A*. *muciniphila* were distinct from the microbiota members *F*. *prausnitzii* A2-165 and *L*. *plantarum* WCFS1. Differential host response towards *A*. *muciniphila* and *F*. *prausnitzii* also became apparent in a study conducted with mouse derived organoids [16]. Therein, *A*. *muciniphila* triggered mostly regulation of metabolic markers as compared to *F*. *prausnitzii*. In the model systems used in the present study *A*. *muciniphila* and *F*. *prausnitzii* live cells induced very similar amounts of IL-10. Interestingly, *A*. *muciniphila* supernatant induced high amounts of IL-10, whereas *F*. *prausnitzii* supernatant barely stimulated any IL-10 production. Furthermore, the TNF-α/IL-10 cytokine induction ratio, which is often used to measure the inflammatory potential of emerging probiotics [35, 36], was lower in *A*. *muciniphila* in comparison to *F*. *prausnitzii* and *L*. *plantarum*. These observations indicate greater anti-inflammatory capacity for both live *A*. *muciniphila* as well as its secreted products and metabolites in comparison to *F*. *prausnitzii* and *L*. *plantarum*.

As Amuc_1100 was shown to be located on the outer membrane of *A*. *muciniphila*, it is plausible that this protein is also liberated to the supernatant and therefore contributes to stimulation of IL-10. Another possibly important difference between the secreted compounds produced by these two bacteria is the production of mucin-degrading enzymes, which are abundantly present in *A*. *muciniphila*, but not in *F*. *prausnitzii*.

The cytokine response of human PBMCs was consistently lower for *L*. *plantarum* compared to *A*. *muciniphila*, except for induction of TNF-α. This is in line with the comparison of mouse transcriptional response to colonization with *A*. *muciniphila* or *L*. *plantarum*, which revealed that *A*. *muciniphila* induces relatively higher up-regulation of genes participating in immune response signaling and ERK/MAPK signaling [15]. Despite greater immune response, the *A*. *muciniphila*-colonized mice did not develop microscopically visible inflammation or show any sign of discomfort. The diverse immune stimulation caused by *A*. *muciniphila* could thus be an indication of gut immune tolerance towards this commensal. The differences between *A*. *muciniphila* and *L*. *plantarum* were also demonstrated in obese and type 2 diabetic mice, where treatment with *A*. *muciniphila* improved the metabolic outcome, whereas treatment with *L*. *plantarum* did not at all have this beneficial effect [17].

The differential immune response between *A*. *muciniphila*, *F*. *prausnitzii* and *L*. *plantarum* could be an indication of their physiological or metabolic differences, or the fact that in the gut these bacteria colonize separate niches. As a mucin-degrader, *A*. *muciniphila* is in closer contact with the host, in contrast to bacteria colonizing the lumen. Our study further strengthens the findings made in the aforementioned studies [15–17] on the variances of host response between these commensal bacteria.

*A*. *muciniphila* LPS gave a strong response with TLR4 and is most likely the activating molecule for this receptor in *A*. *muciniphila*. Recently it has been reported that the position of the phosphate in the lipid A of bacterial LPS may play an important role in separating bacterial-host innate immune system interactions into either symbiotic or pathogenic relationships [37]. Determining the molecular structure of *A*. *muciniphila* LPS would be valuable in understanding its immunostimulatory role in the gut. Previously, it was proposed that LPS of *A*. *muciniphila* is inflammatory in a model of experimental alcoholic liver disease in mice, as the levels of *A*. *muciniphila* were higher after chronic intragastric alcohol feeding, and lower in Muc2^-/-^ mice, along with lower plasma LPS concentration [38].

TLR2 is best known for recognizing lipoteichoic acid (LTA) from Gram-positive bacteria, but some Gram-negative bacteria containing non-classical LPS have also been shown to signal through TLR2 [39, 40]. As the lipid structure of *A*. *muciniphila* membranes is not characterized in detail, we evaluated the ability of *A*. *muciniphila* LPS to stimulate TLR2. Even though live bacteria and the supernatant induced a strong TLR2 response, purified LPS did not.

*A*. *muciniphila* did not activate TLR5, the intestinal receptor for flagellin, reflecting the notion that there are no flagellin genes found in the genome of *A*. *muciniphila* [41]. Only high concentrations of *A*. *muciniphila* (10^7^ bacteria/well) induced a minor response of the TLR9 receptor, which recognizes unmethylated CpG sequences in DNA molecules. Isolated *A*. *muciniphila* DNA did not induce any TLR9 response. The genome of *A*. *muciniphila* has a lower than median frequency (255 vs. 401) of GTCGTT hexamers, in comparison with 59 other bacterial species [42], which may explain the observed low activation of TLR9. Another explanation may be the intracellular location of TLR9, which makes it more difficult for the ligands to reach it, especially in an *in vitro* setting.

The localization of *A*. *muciniphila* in the mucus layer, close to the epithelial layer, probably has had a great impact on the mechanisms of immune modulation this bacterium has developed. As *A*. *muciniphila* is diminished in many inflammatory diseases, it could be speculated that the absence of *A*. *muciniphila* in case of inflammation prevents immune suppression at the mucosal epithelial border. Cross-talk between *A*. *muciniphila* and the host might affect immunological tolerance and homeostasis within the gut, possibly by keeping the immune system alert for potential disruptions. *A*. *muciniphila* has been shown to restore mucus layer thickness and to increase intestinal endocannabinoids in diet-induced obese mice [17], suggesting at the same time it contributes to improving gut barrier function. Here we showed that *A*. *muciniphila* and the outer membrane protein Amuc_1100 increased the development of TEER in Caco2-cells, which also indicates strengthening of the epithelial barrier function. The efficacy of the recombinant Amuc_1100 protein to improve gut barrier and restrain the development of high-fat diet-induced obesity has recently been demonstrated in mice [32].

Altogether, these results could partially explain the positive correlation between levels of *A*. *muciniphila* and gut health. Several studies have reported depletion of *A*. *muciniphila* in the fecal microbiota of ulcerative colitis patients, both in remission [10, 43] and in clinically active disease [44]. In addition to fecal microbiota, reduced levels of *A*. *muciniphila* have also been found in biopsies of intestinal mucosa from IBD-patients in comparison to healthy controls [9].

In conclusion, this study revealed the diverse immunostimulatory capacities of *A*. *muciniphila* and identified candidate bacterial products that mediate this stimulation. We have also shown that established and next-generation probiotics have a wide range of species-specific immune stimulatory properties, which should be taken into consideration when developing new applications and interventions.

## Supporting information

S1 File Supporting information. Fig A. TNF- α/IL-10 cytokine induction ratio. Fig B. Silver staining of *A*. *muciniphila* LPS. Fig C. TLR2 signaling of acetate and propionate. Fig D. TEER development in purified proteins of *A*. *muciniphila*. Table A. PCR-primers used for plasmid construction. Table B. Number of cells seeded for the human HEK-Blue hTLR2/4/5/9/NOD2 cell lines.
(PDF)

S1 Dataset Proteomic analysis of *A. muciniphila* sucrose density-gradient fractions. Results are presented as log10 label-free quantification (LFQ) intensities. Non-existing LFQ intensity values due to not enough quantified peptides were substituted with the value 3.5.
(XLSX)
