# Supplementary material for: Pili-like proteins of Akkermansia muciniphila modulate host immune responses and gut barrier function
Source: PLoS One. 2017 Mar 1;12(3):e0173004. doi: 10.1371/journal.pone.0173004 (PMC5332112; doi:10.1371/journal.pone.0173004)
Supplement: S1 File — Fig A. TNF- α/IL-10 cytokine induction ratio. Fig B. Silver staining of A. muciniphila LPS. Fig C. TLR2 signaling of acetate and propionate. Fig D. TEER development in purified proteins of A. muciniphila. Table A. PCR-primers used for plasmid construction. Table B. Number of cells seeded for the human HEK-Blue hTLR2/4/5/9/NOD2 cell lines. (PDF) [file pone.0173004.s001.pdf]

S1 file

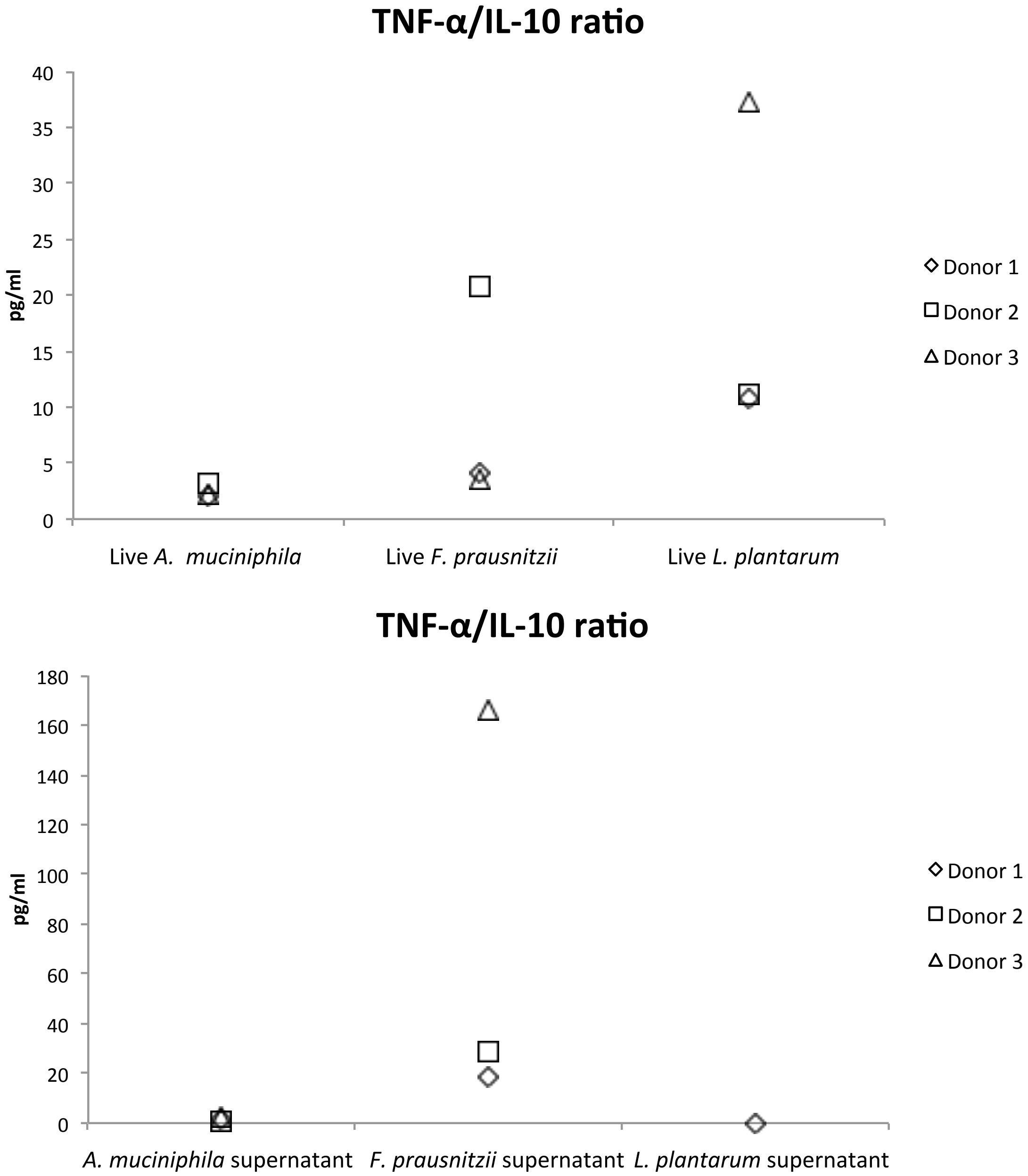

Fig A. TNF- α/IL-10 cytokine induction ratio of *A. muciniphila*, *F. prausnitzii* and *L. plantarum* in human PBMCs for each donor (n = 3).

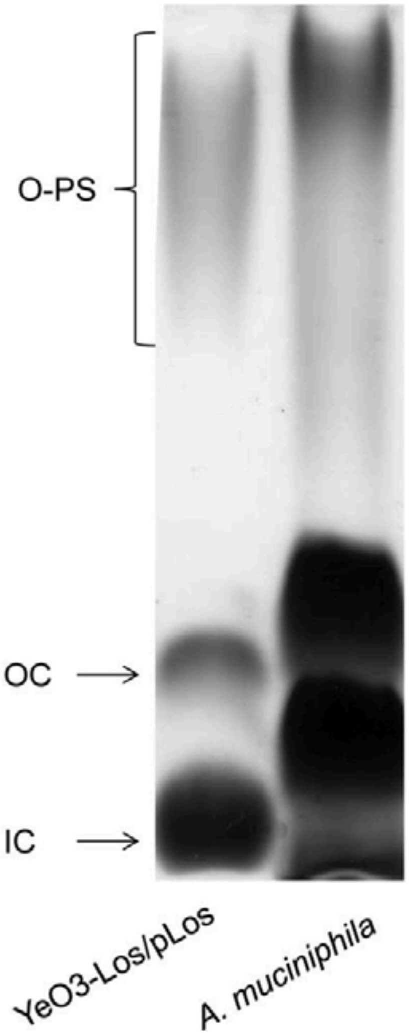

Fig B. Silver staining of *A. muciniphila* LPS. *Yersinia enterocolitica* (strain YeO3-Los/pLos) LPS was used as a control (Pinta et al., 2012). O-PS; O-antigen, OC; outer core, IC; inner core. *A. muciniphila* LPS migrates slower than YeO3-Los/pLos LPS, suggesting the OC and IC bands have in their structure more sugar residues than the corresponding YeO3-Los/pLos bands. Lanes in between the shown samples were removed from the gel image.

Reference:

Pinta, E., Li, Z., Batzilla, J., Pajunen, M., Kasanen, T., Rabsztyn, K., Rakin, A., and Skurnik, M. (2012). Identification of three oligo-/polysaccharide-specific ligases in Yersinia enterocolitica. Molecular microbiology *83*, 125-136.

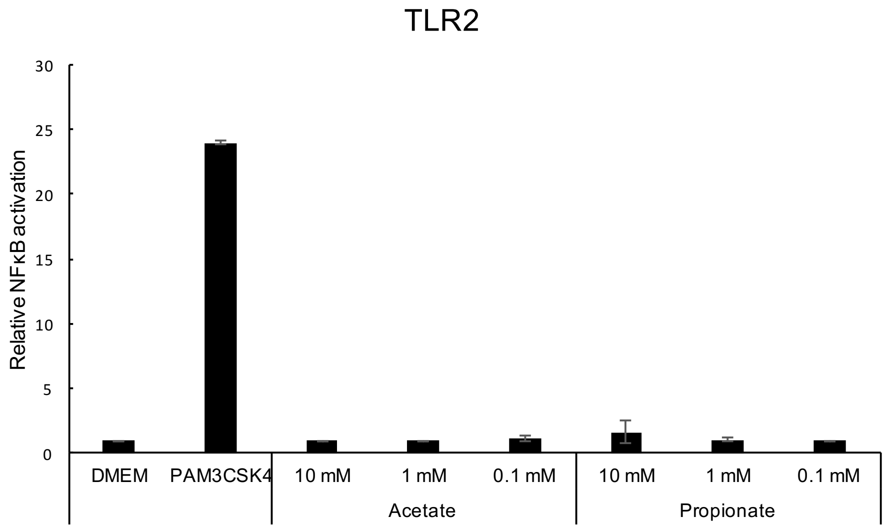

Figure C. TLR2 signaling of acetate and propionate. DMEM; medium control, PAM3CSK4; positive control. (n=3)

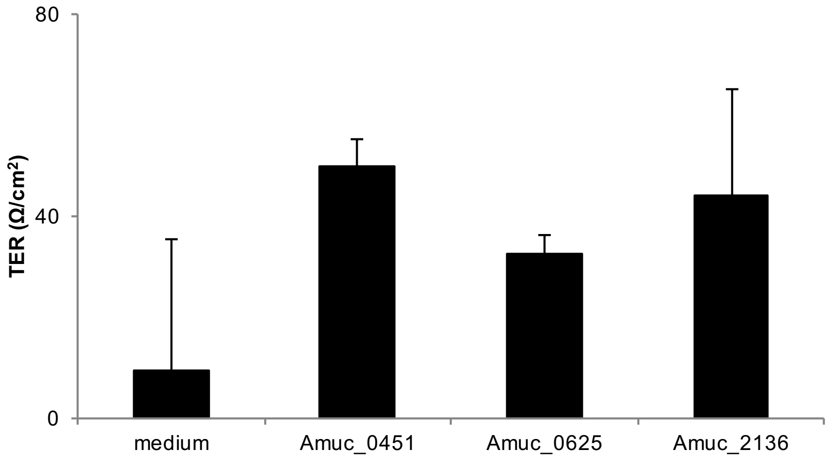

Figure D. The impact of purified proteins Amuc_0451 (0.05 µg/ml), Amuc_0625 (0.05 µg/ml) and Amuc_2136 (0.05 µg/ml) on the TEER development of Caco-2 monolayer after 24 h of stimulation. Mean and standard deviations from three parallel wells are shown.

Table A. PCR-primers used for plasmid construction.

Gene	Forward primer sequence (5' - 3')*	Reverse primer sequence (5' - 3')*
**Amuc_0451**	gcagcgcatatgaagccggctgcccggtctgtcaaaact	ggacgcctcgagcctggattcctt
**Amuc_0625**	gggcagcatatggaagagaaaaccggtttccctacg	ggctttcctctcgagcttgagaacaggagc
**Amuc_1100**	gggtaccatatgatcgtcaattccaaacgc	ccttggctcgagatcttcagacggttcctg
**Amuc_2136**	gatgacatgtccgacgccaagcagattgcggattccctt	catctcgagcaacggctgtacgttcat

* Restriction enzyme recognition sequences are underlined

Table B. Number of cells seeded for the human HEK-Blue hTLR2/4/5/9/NOD2 cell lines.

Cell line	Cells/well
**TLR2**	50000
**TLR4**	25000
**TLR5**	25000
**TLR9**	80000
**NOD2**	25000
